# The ethics of machine learning-based clinical decision support: an analysis through the lens of professionalisation theory

**DOI:** 10.1186/s12910-021-00679-3

**Published:** 2021-08-19

**Authors:** Nils B. Heyen, Sabine Salloch

**Affiliations:** 1 Competence Center Emerging Technologies, Fraunhofer Institute for Systems and Innovation Research ISI, Breslauer Str. 48, 76139 Karlsruhe, Germany; 2 Institute of Ethics, History and Philosophy of Medicine, Hannover Medical School, Carl-Neuberg-Str. 1, 30625 Hannover, Germany

**Keywords:** Clinical decision support systems, Machine learning, Algorithms, Artificial intelligence, Ethics, Professionalisation, Profession, Physicians, Patient autonomy, Physician–patient relationship

## Abstract

**Background:**

Machine learning-based clinical decision support systems (ML_CDSS) are increasingly employed in various sectors of health care aiming at supporting clinicians’ practice by matching the characteristics of individual patients with a computerised clinical knowledge base. Some studies even indicate that ML_CDSS may surpass physicians’ competencies regarding specific isolated tasks. From an ethical perspective, however, the usage of ML_CDSS in medical practice touches on a range of fundamental normative issues. This article aims to add to the ethical discussion by using professionalisation theory as an analytical lens for investigating how medical action at the micro level and the physician–patient relationship might be affected by the employment of ML_CDSS.

**Main text:**

Professionalisation theory, as a distinct sociological framework, provides an elaborated account of what constitutes client-related professional action, such as medical action, at its core and why it is more than pure expertise-based action. Professionalisation theory is introduced by presenting five general structural features of professionalised medical practice: (i) the patient has a concern; (ii) the physician deals with the patient’s concern; (iii) s/he gives assistance without patronising; (iv) s/he regards the patient in a holistic manner without building up a private relationship; and (v) s/he applies her/his general expertise to the particularities of the individual case. Each of these five key aspects are then analysed regarding the usage of ML_CDSS, thereby integrating the perspectives of professionalisation theory and medical ethics.

**Conclusions:**

Using ML_CDSS in medical practice requires the physician to pay special attention to those facts of the individual case that cannot be comprehensively considered by ML_CDSS, for example, the patient’s personality, life situation or cultural background. Moreover, the more routinized the use of ML_CDSS becomes in clinical practice, the more that physicians need to focus on the patient’s concern and strengthen patient autonomy, for instance, by adequately integrating digital decision support in shared decision-making.

## Background

Machine learning (ML) applications are increasingly employed in various sectors of health care and accompanied by the promise of making patient care more effective, reliable and affordable. In contrast to traditional forms of computer programming, ML relies on data-driven rules which are derived from large datasets rather than being fully specified in advance by a human programmer. Based on such ‘training data’, ML applications can make predictions, guide decisions and automatically improve through their own experience. Machine learning is often used within health care as a technological background for clinical decision support systems (CDSS), which serve as a direct aid to clinical decision-making and aim at supporting clinicians’ practice by matching the characteristics of individual patients with a computerised clinical knowledge database [1]. The scope of ML-based CDSS (ML_CDSS) is considerably broad. Various branches of medical imaging, for example, benefit potentially from ML applications when dealing with complex tasks, such as object classification, detection and segmentation [2–4]. Clinical diagnostics, for example, in ophthalmology, can also be supported significantly by methods of advanced data science [4, 5]. In addition to the application of ML in diagnostics, there are also promising approaches in such different fields as robotic-assisted surgery [6], human genomics [7, 8] or prevention [9, 10].

From an ethical perspective, the usage of ML (particularly systems operating with deep learning and artificial neural networks) for health-care purposes touches on a range of fundamental normative concepts, such as agency, trustworthiness, transparency and responsibility [11]. A recent mapping review of the literature on the ethics of artificial intelligence (AI) in health care found that such issues have a considerably broad scope and arise on six levels of abstraction: the individual, interpersonal, group, institutional, societal and sectoral level [12]. Major points of the ethical discussion on ML applications in health care are closely linked to fundamental epistemic issues, such as inconclusive, inscrutable or misguided evidence, which lead to normative concerns, such as unfair outcomes or transformative effects, related to privacy and individual identity [13]. Consequently, systematic frameworks have been suggested for dealing comprehensively with the ethics of ML applications in health care [14]. Moreover, the ethics of ML generally [15] and particularly in health care has been the subject of a multitude of national and international guidelines and recommendation papers [16].

This contribution aims to add to the ethical discussion about the impact of ML_CDSS on medical practice by setting out from the viewpoint of professionalisation theory as a distinct sociological framework. Professionalisation theory provides us with an elaborated account of what constitutes client-related professional action, such as medical action, at its core, and why it is more than pure expertise-based action. We take this theoretical approach as an analytical lens for investigating how medical practice at the micro level is affected by the increasing employment of ML technologies and how professionals might react and deal with these technologies in relation to the patients from an ethical perspective. A ‘full picture’ of the interaction between ML_CDSS and medical professionalism would also need to consider institutional and systemic aspects of health care. Our approach is limited to the micro perspective of patient–physician interaction because a more comprehensive analysis could not be adequately dealt with within one article. Methodologically, the article draws on an established approach from sociological professionalisation theory which puts special emphasis on the way in which professionals deal with clients’ concerns. Ethical issues related to ML_CDSS are highlighted in so far as they become apparent when taking a closer look at professional action within the patient–physician encounter. The article, therefore, does not aim to provide a full analysis of the ethics of ML_CDSS but is restricted to those issues which arise from a closer examination of professionalism from a distinct and elaborated theoretical approach. As a result, the article might contribute to a fuller understanding of the ethical consequences arising from the impact of ML_CDSS on the professional role of physicians.

In the early sections of our article, we briefly contextualize the employment of ML_CDSS within a broader medical and ethical discourse and take a look at the performance of ML-based applications in comparison to medical experts. We then introduce professionalisation theory by presenting five general structural features of a professionalised medical practice. Subsequently, we reflect on each of these five key aspects regarding the use of ML_CDSS, thereby integrating the perspectives of professionalisation theory and medical ethics. We end with some conclusions and tentative recommendations.

## Main text

### Men or machines?

Recent discussions on the introduction of advanced data science in clinical practice and its impact on the physician’s role are situated in a wider discursive context which considers digitisation as a key aspect in the development of contemporary societies. The ML applications are not only increasingly present in health care but also in various other branches, such as management, jurisprudence, journalism and architecture. Advanced computer technology in many knowledge-intensive sectors has already helped to deal with complex tasks which had previously typically been the subject of human expertise. Academic jobs are, thus, not exempt from digitisation but today stand right at the centre of being supported (or partly replaced) by systems of so-called AI. Whereas techno-optimist positions welcome the increasing usage of computational systems at the workplace [17, 18], more sceptical authors warn of an ‘AI takeover’ which could challenge human expertise and competence [19].

The awakening interest of the medical profession in ML_CDSS has been raised, not least due to the emergence of studies comparing physicians’ clinical skills with the performance of ML-based applications. The—mainly visual—competence of a dermatologist in classifying skin lesions, for example, has been shown to be comparable with the respective performance of an automated classification system running with deep convolutional neural networks. A deep convolutional neural network was trained with about 130,000 clinical images of skin lesions in a comparative trial [20]. The model’s performance was then tested against 21 board-certified dermatologists in classifying images of benign versus malign lesions. Consequently, the deep convolutional neural network outperformed most of the clinicians regarding the sensitivity and specificity of the diagnoses. Other studies from dermatology operating with similar methods show that convolutional neural networks surpass clinicians’ diagnostic performance particularly regarding the specificity of the results [21]. Similar promising findings for the use of ML in diagnostics have been derived concerning chest X-ray evaluation [22] and arrhythmia detection in electrocardiograms [23]. More complicated diagnostic tasks have also been the subject of trials comparing the performance of clinicians and algorithms. Liang et al., for example, applied ML classifiers to about 1.3 million electronic health records from paediatric patients [24]. An automated natural language processing system applied deep learning techniques to extract clinically relevant information and was trained to imitate physicians’ hypothetico-deductive reasoning. The model’s performance was compared to the diagnoses of 20 paediatricians of varying professional experience. Consequently, the average score achieved by the model was higher than in the junior physician groups but lower than in the senior physician groups.

Methodological details (e.g. the sample size, composition of the control group or ecological validity) of such comparative studies demand further scrutiny and the results cannot be easily generalised to other diagnostic branches or clinical practice so far. The discussion on the ethical implications of integrating ML_CDSS at the physician’s workplace, however, is already in full swing [11, 25–27]. Similar to digitisation in general, positions regarding the future of the medical profession in digitised health care are markedly divided to date: optimist positions highlight the potential of advanced data science for promoting a personalised, patient-centred or even more humane patient care [28]. According to this view, technical support systems, for example, could be applied for creating better options for physicians to invest in relational and empathetic aspects of their practice. By contrast, other authors stress that ML systems have not yet proven their positive effects on health outcomes and may cause unwanted effects on professional practice, such as an over-reliance on computers, deskilling or a loss of confidence in providing diagnoses [29].

Against this background, it is not surprising that physicians’ institutional bodies have tackled the issue of digital decision support intensively in scientific conferences and events, statements and opinion papers [30, 31]. In addition, reference to the ‘profession’ is widespread in the current discourse on the digitisation of health care [25], whereas a more elaborate way of dealing with medical professionalism and professionalisation theory has so far been missing. This article, therefore, takes up the thread of the current discourse on the ethical implications of ML_CDSS and aims to add a systematic analysis through the lens of professionalisation theory.

### Professionalisation theory

Professions and professional action have been the subject of sociological investigation for almost a century (e.g. [32, 33]). Many scholars have been concerned with the institutional and organisational characteristics and dynamics of professions on a macro level (e.g. [34–37]). By contrast, others, particularly the German-speaking sociology of professions (building on classical works such as [32, 33, 38, 39]), have focused on the micro level, thus, the structural features of professional action in general. Regardless of the various theoretical approaches, it has been widely recognised that, in simple terms, professional action generally deals with practical problems or crises of people (clients or patients) and usually draws on scientific knowledge. However, it cannot simply apply this expertise in terms of schematic rules because it is confronted with contradictory requirements and uncertainties of various kinds.

In order to avoid misunderstandings, it has to be stressed that the counterpart of the role of the professional has been called a ‘client’ since the beginnings of the sociology of professions [32]. The client is the person who engages professional advice or service. In common parlance, this term is used, for instance, regarding the role pair of lawyer and client. A client in this sense is neither a customer nor a consumer, both known from economic contexts. Thus, wherever we speak of a client as the counterpart of the medical professional in this article, we mean nothing else than the classic role of the patient. Furthermore, as will become clear below, from the perspective of professionalisation theory, a reduction of the role of the patient to something like a customer or consumer (as it has been empirically observed and ethically criticised in the context of an economisation of health care) would be a clear sign of *de*professionalisation.

The ideal type of client-related professionalised action has been specified and tested in numerous empirical studies of medical practice in the professionalisation theory developed by Oevermann [40, 41]. According to this elaborated approach, a professionalised medical practice is characterised by five structural features [42]:

(i) *The patient has a concern* resulting from a problem or crisis that affects her or his everyday life and that she or he cannot solve alone or autonomously. Therefore, the patient seeks help from a professional. Although this seems self-evident, not being in need of help or advice is a bad prerequisite for professional action and may cause difficulties.
(ii) The task of the physician is to cope with or solve the problem or crisis of the patient vicariously. Thus, *the physician deals with the patient’s concern* or problem. Again, this seems self-evident, but it is important that the physician works, in fact, on the patient’s concern, whatever it might be, and not on something else that the professional might consider to be important or the ‘real’ issue.
(iii) The physician deals with the patient’s concern *together with the patient*. The patient can neither simply leave her or his problem (or sick body) at the counter like a defective device, nor can the physician solve the problem without the patient. Therefore, both physician and patient must enter into a working alliance in order to tackle the problem in focus together. By doing so, the physician is confronted with a general tension that she or he has to withstand rather than resolve unilaterally. That is the tension between the patient’s *autonomy and dependency* vis-à-vis the professional. On the one hand, the patient might place her/himself in a particularly pronounced dependency, for instance, on the operating physician. On the other hand, the patient has to autonomously consent to this relinquishment of autonomy. Therefore, *the physician gives assistance without patronising*.
(iv) In addition, *the physician regards the patient in a holistic manner without building up a private relationship*. This concerns another tension, namely, between the *diffusivity and role-relatedness* of the physician–patient relationship. On the one hand, the patient approaches the doctor as a ‘whole person’, thus, not bound to a specific role but rather diffuse, as in family or friendly relationships. Indeed, everything about the patient and her or his history can be interesting or necessary for the physician, even intimate details from the patient’s private life. On the other hand, this diffusivity also has its limits. The patient always remains relevant in the role of a service recipient. Thus, the physician is interested in the patient’s personal matters but only insofar as they are relevant to the physician’s professional task of providing medical assistance and not out of curiosity or personal interest.
(v) Finally, *the physician applies her or his general expertise to the particularities of the individual case*. This addresses a third tension, namely, between the *knowledge base and case specificity*. An individual case is always a ‘case of X’, thus, a representation of a general structure or relationship [43]. It is the task of the professional to find an understanding of the patient’s problem which is, on the one hand, adequate to her or his general medico-scientific knowledge and gives, on the other hand, expression to the particularities of the patient’s individual problem situation.

Due to the three tensions outlined above (autonomy vs. dependency, diffusivity vs. role-relatedness and knowledge base vs. case specificity), professionalisation theory does not consider professional medical action to be essentially standardizable. This does not mean that there are or should not be standards of ‘good medical practice’ and corresponding guidelines. However, these cannot be applied in a schematic, formalistic way. Conversely, the key characteristic of professionalised action is to withstand the three tensions. Resolving them unilaterally would mean to act in a non-professionalised manner.

### Ethics of ML_CDSS from the perspective of professionalisation theory

In this section, we take the five structural features of professionalised medical practice introduced above as an analytical lens for gaining a closer look at the use of ML_CDSS in medical practice. We reflect on the five structural features separately, in each case integrating the perspective of professionalisation theory with a discussion of ethical implications. We presuppose in our analysis that the ML_CDSS is integrated into the physician’s workflow and does not interact directly with the patient. Patient-side ML applications (such as symptom checkers or fitness apps) are, thus, not considered even if they are similarly associated with important ethical issues in light of professionalisation theory.

(i) *The patient has a concern.* This also holds true, of course, in a medical practice integrating ML_CDSS. However, it might be less clear, particularly in practices dealing with prevention, whether or to what extent a patient has a concern at all. Here, an increasing routinization, most notably in highly specialised clinical institutions, of clarifying whether specific risk dispositions are existent or not runs the danger of ignoring the question to what extent such a clarification is really in the patient’s interest. The general availability of an ML_CDSS may, thus, enforce a physician’s attitude to routinely feed the ML_CDSS with the patient data at hand without engaging in building up a sound and professional physician–patient relationship by asking, for instance, the initial question “What is your concern?” or “How can I help you?”.

These initial and relationship-building questions are also crucial for scrutinizing whether the special expertise of the physician is appropriate to tackle the patient’s concern and, more generally, whether it is actually a *medical* issue that motivates the patient to seek the physician’s help and not a problem originating, for example, solely from the job or private situation. The availability of technological resources and the increasing inclusion of information other than biomedical data (e.g. data on mobility, nutrition or from social media) increasingly blurs the border between the medical sphere and ‘private life’ and might prompt the physician to deal with issues that do not lie within the scope of her or his competency. If the patient’s concern does not fit the physician’s field of expertise, it is the task of the latter to point out this incongruity and, ideally, refer the patient to another, more appropriate professional field.

(ii) *The physician deals with the patient’s concern*. Taking this seriously, it means that the physician uses the ML_CDSS only and exclusively if this is motivated by the patient’s concern. At least two questions arise here: are other (legitimate) interests influencing the physician’s use of the ML_CDSS and do additional issues arise from the use of the ML_CDSS which go beyond the patient’s initial interest?

Regarding the first question, an ethically reflective employment of ML_CDSS should consider that the enormous technological options inherent in these systems might lead to forms of employment which deviate from dealing with the patient’s concern in a strict sense. An obvious field of controversy lies in the intersection between clinical practice and research. It has already been highlighted that the borders between these two fields become increasingly unclear in the use of ML_CDSS [11]. As the machines continue to develop in their use (‘learning’), patients are typically feeding their data and contribute to a training and refinement of the algorithmic tools. Whereas it can be considered as an ethical imperative to continuously develop the quality of learning health-care systems [44], the blurred boundaries between clinical care and research might place high requirements on patient information and could prevent physicians from taking measures which are directed exclusively to the individual patient’s welfare. Well-established guidelines exist on how to deal with conflicts of interest, for example, in clinical studies or guideline development [45]. The situation seems to be less clear, however, regarding potential institutional directives or economic incentives to use or test an ML_CDSS in practice and let it develop further based on patient data.

The other question concerns, for example, the occurrence of secondary findings which were not targeted by the diagnostics and, thus, do not necessarily mirror the patient’s concern. Secondary findings arising from ML_CDSS might extend, for instance, to risk predictions of various adverse events (such as death or cardiovascular complications) or findings about the patient’s supposed lifestyle or therapeutic adherence. From the perspective of professionalisation theory and an ethical perspective, the eventuality of secondary findings and how the physician is supposed to deal with them should be discussed and reflected on together with the patient. Only if the patient includes such further findings (at best specified) in her or his concern before the use of the ML_CDSS does the physician receive the mandate to later reveal and share them with the patient.

(iii) *While dealing with the patient’s concern, the physician gives assistance without patronising (tension: autonomy vs. dependency).* Regarding ML_CDSS, there are several important aspects to discuss. Firstly, if the use of an ML_CDSS implies any risks to the diagnostic or therapeutic process, these risks need to be made transparent and discussed with the patient. Such risks may include a high rate of false positive or false negative results, or problematic secondary findings, as discussed above. In the case of truly severe risks associated with ML_CDSS and approved available alternative procedures, such alternatives should be offered, discussed and made accessible to the patient. Particularly regarding secondary findings, it could appear to be helpful to learn from other fields, such as medical genetics and genetic counselling, a highly professionalised practice [42] where the patient’s ‘right not to know’ has been intensively discussed and thoroughly implemented [46]. Unilaterally resolving the tension between the patient’s autonomy and dependency (here, towards dependency) would mean concealing any risk or other information that might restrain the patient from consenting or adhering to the use of an ML_CDSS.

Another important aspect concerns, again, the degree of routinization of the ML_CDSS. The more routinized or automatically (in the sense of unquestioned) the physician follows the output or recommendations of an ML_CDSS, the higher the patient’s dependency on this ML_CDSS. Therefore, the physician needs to compensate this ML_CDSS-dependency by strengthening the patient’s autonomy. This might be realised, for example, by explaining the functionality of the ML_CDSS and potential shortcomings, pointing to alternative interpretations that complement the ML_CDSS output and supporting the patient in finding a decision regarding all reasonable options for further treatment, if applicable. It has to be noted, however, that attempts to increase the patients’ digital health literacy [47] might remain limited in light of the complexity and opacity of advanced ML technologies. Patient education is, thus, closely related here to technological challenges of explainability in advanced data science [48]. Nevertheless, unilaterally resolving the tension between the patient’s autonomy and dependency (here again, towards dependency) would mean to follow any recommendation of an ML_CDSS in a unreflected way and, simultaneously, to refrain from problematising the ML_CDSS output vis-à-vis the patient, even if there are good reasons to do so.

Such an impact of ML_CDSS on patient autonomy can also be discussed in the light of a ‘computer-paternalism’ (“Computer knows best”), which might become apparent when AI systems recommend and rank treatment options without considering the individual patient’s values and preferences [49]. The ML_CDSS might then be depicted as exhibiting a patronising ‘attitude’ towards the patient. Bioethicists, therefore, plead for a value-flexible design of AI systems [49] and a meaningful and reflective integration of these supporting systems into the physician–patient relationship [50].

Further issues in the intersection between ML_CDSS, patient autonomy and dependency include the question whether patients should have the right to refrain from the use of AI systems and, instead, to stick to the less advanced alternatives. On the one hand, it can be sensibly argued that patients should be able to withdraw from AI diagnostics and treatment due to the specific role of the physician, bias and opacity problems, or the future impact of AI systems on the health-care system [51]. On the other hand, health-care systems could rapidly become strained if they need to hold available both alternatives in the future: medical technologies based on advanced data science and their ‘analogous equivalents’. If ML_CDSS increasingly prove to be effective and secure, there need to be good reasons to grant ‘opt-out’ rights to patients as there is generally no patient right to stick to outdated procedures in health care. In addition, from the point of view of professionalisation theory, preserving the patient’s autonomy means to enable her or him to choose between alternative, justifiable options. However, leaving the decision to the patient alone without providing information, giving advice or supporting her/him in finding the individual ‘right’ decision would similarly resolve the tension between the patient’s autonomy and dependency in a unilateral way (but this time, towards autonomy; the term ‘autonomy’ here is not used in the bioethical sense but from the viewpoint of professionalisation theory; it could, in this case, be translated to ‘independency’).

(iv) *The physician regards the patient in a holistic manner without building up a private relationship (tension: diffusivity vs. role-relatedness).* Using an ML_CDSS in medical practice entails the risk of ignoring patient-related information and aspects that are, in fact, relevant from a holistic medical perspective but cannot be fed as data into the ML_CDSS. To take a rather simple example, this may concern the right timing of an invasive intervention in relation to the general life situation of the patient, such as the current employment or family situation. Thus, the greater the importance of the ML_CDSS within the professional decision-making process, the more the physician needs to make sure that all aspects of the patient’s life situation that might be relevant for coping with the health problem and for a good health outcome are laid on the table and considered appropriately. Not taking such aspects into account would unilaterally resolve the tension between the diffusivity and role-relatedness of the physician–patient relationship (here, towards role-relatedness), as the patient is reduced to the role of a mere data provider.

On the other hand, even if the attempt to treat the patient holistically also causes a physician to consider ‘non-biomedical’ facts (such as patient’s life situation, outward appearance or behavior during the conversation), she or he must consistently stick to the professional role and should not unduly (i.e. without a good reason) intrude into the patient’s privacy. With the increasing use of ML_CDSS, the tension between diffusivity and role-relatedness is intensifying even more due to several factors: the widespread deployment of ML technologies for preventive purposes leads to a situation where the clinician might get insights into aspects of the patient’s life (e.g. mobility, nutrition, sleep habits) which could—or could not—be relevant to medical practice. The increasing use of data from non-medical contexts (e.g. social media data for psychiatric diagnostics [52]) leads to an extension of the medical sphere to fields in life which have previously had a non-medical character. Significant chances and challenges (e.g. emerging from the expansion of the concept of disease [53]), therefore, may arise from the use of ML_CDSS regarding the aim of treating patients in a holistic manner. Unilaterally resolving the tension between the diffusivity and role-relatedness of the physician–patient relationship (here, towards diffusivity) would mean to make use of such non-medical data for purposes that do no fall within the physician’s scope of responsibility.

(v) *The physician applies her or his general expertise to the particularities of the individual case (tension: knowledge base vs. case specificity).* This aspect once again underlines that the physician has to ensure that information on the individual patient that cannot technically be included in the ML_CDSS is taken into account within the professional decision-making process. The more important and routinized the use of an ML_CDSS is, the more the physician needs to scrutinize whether the ML_CDSS output is still valid regarding the individual case, including all relevant information available. Ignoring such case-specific information would unilaterally resolve the tension between the knowledge base and case specificity (here, towards the general but limited knowledge base of the ML_CDSS).

In addition, this tension points to the matter of trust (in the ML_CDSS), an issue widely discussed in the literature on ML and AI in general, including trustworthy or explainable AI [54, 55]. The physician needs to be able to trust (and periodically reaffirm) that the ML_CDSS represents (or outperforms) her or his own medico-scientific knowledge adequately and that its use improves the outcome of her or his medical practice. The more this is uncertain, the less the physician should rely on the ML_CDSS. Still relying on the technical system, despite existing uncertainty or doubt, would again unilaterally resolve the tension between the knowledge base and case specificity (here again, towards the knowledge base of the ML_CDSS). The reverse case, resolving the tension towards case specificity, would mean to get lost in the particularities of the individual case and neglect to relate them in a meaningful way to the general knowledge base of the ML_CDSS.

The question to what extent ML_CDSS are equipped for dealing with individual cases, finally leads to a particularly challenging aspect in the ethical evaluation of such systems. As outlined above, recent studies indicate that AI systems meet or even surpass physicians’ competencies regarding specific, isolated tasks. This punctual superiority is likely to grow in the near future as the technological advancement progresses. However, it becomes increasingly obvious that automated procedures of ML (that is part of the knowledge base from the perspective of professionalisation theory) can promote biases and disadvantage certain groups of patients who have not, for example, been adequately represented in the training data. In addition, based on the training data, algorithmic biases may reproduce, for instance, racial biases already present in health-care practice [56, 57]. The application of ML in health care, therefore, needs permanent surveillance because incorporating a particular practice into an algorithm may imply an unsubstantiated legitimacy of that practice which is not justified by an improved outcome for the individual patient [58]. Whereas ML_CDSS might give rise to the impression that subjective human judgement is replaced with unadulterated data-driven recommendations, in fact, ‘new’ forms of discrimination can arise within data technologies, for example, when labelling or annotating the data. Two aspects need to be considered when using a big data knowledge base for the care of individual patients: the risk of perpetuating wrongful clinical practices inherent in the training data and illegitimate discrimination arising from the algorithmic nature of the decision support itself.

## Conclusions

Taking professionalisation theory as an analytical framework allows one to take a fresh look at the ethical implications of ML_CDSS and sheds light on several aspects that have been neglected in the scientific discussion so far. The specific focus on the physician’s commitment to the patient’s concern, for example, should be taken as a strong signal which must not be overlooked when applying ML_CDSS in clinical practice. In addition, the aspect of role-relatedness—as taken from professionalisation theory—exemplifies how ML_CDSS tend to transgress what has traditionally been considered the ‘medical sphere’ and enters into fields which had previously been ‘non-medical’. Physicians should be aware of this new kind of information source, and the medical profession should reflect how to deal with this extension of the physicians’ scope of activities, which can be easily linked to ethically significant concepts, such as medicalisation. Another aspect relates to the non-standardizability of professional action which results from the demand to withstand the three tensions discussed above and to not unilaterally resolve them in one or the other direction. That necessarily implies the alignment to individual medical cases. Although such a case specificity might also be realised via the application of ML-CDSS, the corresponding limits of these systems due to their given database should not be overlooked. The way of ‘personalising’ diagnostics and treatment may generally differ considerably between human medical experts and machines.

When using ML_CDSS in medical practice, special attention must, therefore, be directed to the ‘soft’ facts of the individual case which cannot be comprehensively considered by ML_CDSS, for example, the patient’s personality, life situation or cultural background. The more routinized the use of ML_CDSS becomes in clinical practice, the more physicians need to focus on the patient’s concern and strengthen patient autonomy, for example, by adequately integrating digital decision support in shared decision-making together with the patient.

In more general terms of professionalisation theory, the usage of ML_CDSS in medical practice must not lead to a unilateral resolving of any of the three tensions discussed in this article (autonomy vs. dependency, diffusivity vs. role-relatedness and knowledge base vs. case specificity). Therefore, whenever specific aspects or steps of the diagnostic or other decision process tend to be left to an ML_CDSS, the implications (gains and losses as well as options for compensating these losses) need to be thoroughly reflected on not only by the professional institutional bodies but also by each individual physician who applies such systems in her or his medical practice. Major aims for the future development and clinical implementation of ML_CDSS should, therefore, lie in physicians’ education and reflection on digital decision support and in a reasonable distribution of tasks which considers the strengths and limitations of both decision support systems and human doctors.

As our first attempt regarding an interdisciplinary and integrated analysis of professional and ethical aspects of ML_CDSS has yielded promising results, we would encourage conducting further research in this direction—conceptual and empirical—and engaging in co-operations with other academic disciplines for comprehensive analyses of digital decision support. Meso and macro perspectives on medical professionalism and their impact on the patient–physician relationship should also be considered in future research. In addition to ethical and sociological perspectives, expertise is needed, for example, from the technical disciplines (including human–machine interaction), clinical medicine and jurisprudence. Joining these scientific and professional forces may contribute further to the exploration and understanding of the prerequisites of a responsible and trustworthy use of ML_CDSS and other AI-based systems in professionalised medical practice.

## Data Availability

Not applicable.

## Abbreviations

AI: Artificial intelligence
CDSS: Clinical decision support systems
ML: Machine learning
ML_CDSS: ML-based CDSS
